# Early tumor shrinkage correlates with final EBRT response in cervical cancer patients treated with MRI-guided radiotherapy

**DOI:** 10.1016/j.ctro.2026.101158

**Published:** 2026-04-06

**Authors:** Perla Zidane, Thomas Berger, Angela Boros, Ionela Caraivan, Mazen Moussallem, Corina Udrescu, Olivier Chapet

**Affiliations:** a Department of Radiation Oncology, Centre hospitalier Lyon Sud, Hospices Civils de Lyon, Pierre-Benite, France; b Université de Lyon, Université Claude Bernard Lyon 1, Lyon, France; c Holy Family University, Batroun, Lebanon; d Healthy Innovations, Rachiine, Zgharta, Lebanon

**Keywords:** MR-guided radiotherapy, Cervical cancer, Tumor volume evolution

## Abstract

**Background/Purpose:**

Tumor response can vary significantly in locally advanced cervical cancer patients undergoing radiotherapy. Personalizing treatment based on this variability may improve therapeutic outcomes. This study quantified daily tumor volume evolution on MR-Linac and explored its potential in predicting treatment outcome.

**Materials/Methods:**

Sixteen patients were retrospectively analyzed. Daily T2-weighted MRI scans (n = 400) were used to delineate tumor volumes, validated by two radiation oncologists. Tumor volume reduction rates (TVRR) and regression slopes were assessed across fractions. Spearman analysis evaluated correlations with final external beam radiotherapy (EBRT) response. To assess the impact of treatment interruptions, regression slopes were compared between consecutive (1-day interval) and interrupted delivery (>1 day) across early, middle, and late phases.

**Results:**

Median tumor volume decreased from 26.2 cc at baseline to 0.7 cc at fraction 25. TVRR showed progressively stronger associations with end-of-EBRT response, with Spearman coefficients increasing from rho = 0.20 (p = 0.45) at fraction 5 to a peak of rho = 0.67 (p = 0.004) at fraction 10. All patients achieving > 50% reduction by fraction 10 reached > 90% reduction by the end of EBRT. Treatment interruptions were associated with significantly slower regression kinetics, particularly during the early phase (p = 0.003).

**Conclusions:**

Early tumor reduction by fraction 10 is strongly associated with final EBRT response, supporting its role as a potential predictive marker for adaptive strategies. Furthermore, treatment continuity appears critical for optimal regression kinetics in the initial phase. Validation in larger cohorts is needed.

## Highlights

• Early tumor shrinkage predicts final response to external beam radiotherapy.
• ≥50% volume reduction by fraction 10 was associated with ≥ 90% final reduction.
• Treatment interruptions were linked to slower early tumor regression.

## Introduction

Cervical cancer remains the fourth most common malignancy among women worldwide [1]. It poses significant challenges to public health, this is particularly the case in regions with limited access to screening and preventive measures [2]. Survival rates depend mainly on the cancer stage at the time of diagnosis, with locally advanced cervical cancer (LACC), defined as International Federation of Gynecology and Obstetrics (FIGO) stages IB3–IVA (FIGO 2018), representing a significant therapeutic challenge [3].

Standard treatment for LACC includes external beam radiotherapy (EBRT) with concurrent chemotherapy followed by brachytherapy [4], [5]. More recently, the addition of immunotherapy to local treatment for high-risk LACC patients has shown significant improvement in overall survival [6]. Despite the use of modern approaches such as intensity modulated radiotherapy (IMRT) and MRI-guided brachytherapy [5], response to treatment may significantly differ among patients [7], [8], [9], highlighting the need for more personalized management strategies. Identifying radiosensitive versus radioresistant tumors early on could enable adaptive interventions, thereby improving treatment efficacy while minimizing side effects [10].

The Unity® 1.5 T MR-Linac system (Elekta AB, Stockholm, Sweden) integrates a high-field 1.5 T MRI scanner manufactured by Philips (Philips Medical Systems, Best, The Netherlands) with a 7 MV linear accelerator. This enables high-resolution anatomical images to be acquired at each treatment fraction [11] with very high soft tissue contrast, allowing real-time visualization of the tumor and surrounding tissues. The Unity MR-Linac enables daily online adaptive radiotherapy workflows and allows modifications based on patient-specific anatomical changes.

Only a few studies found in the literature studied the tumor evolution [12], [13], [14], [15]. However, currently, there is a lack of detailed analyses of daily tumor evolution and individual response dynamics variations across patients. In this descriptive study, daily tumor volume changes throughout the treatment were characterized in patients with LACC treated on the Unity MR-Linac. The aim was to explore inter-patient variability in tumor shrinkage patterns and to assess whether early volumetric response could serve as a potential biomarker for treatment personalization in the future.

## Materials and methods

### Patient and treatment characteristics

Patients were retrospectively included from a consecutive cohort according to the following criteria: histologically confirmed cervical cancer; completion of radiotherapy treatment on the Unity® MR-Linac system; and no prior history of gynecological radiotherapy to avoid biases in treatment outcomes.

Patients were excluded if they underwent only sequential external beam boost irradiation on the Unity® MR-Linac, if they had prior gynecological radiotherapy, or if they did not complete the planned treatment. The clinical and treatment data were analyzed retrospectively. This retrospective study was approved by the institutional review board. Patients were informed about the study and could decline participation.

All patients underwent EBRT using IMRT to deliver 45 Gy in 25 fractions. In cases of pelvic node involvement, a 55 Gy simultaneous integrated boost (SIB) was administered. Concurrent chemoradiation with weekly cisplatin (40 mg/m^2^) with 2–6 cycles was delivered depending on clinical tolerance. None of the patients included in this study received immunotherapy. A standardized bladder filling protocol was employed throughout the treatment course: patients were instructed to empty their bladder and drink 500 mL of water one hour before every treatment session to reduce interfactional anatomical variability.

### Tumor volume definition

**Fig. 1:**
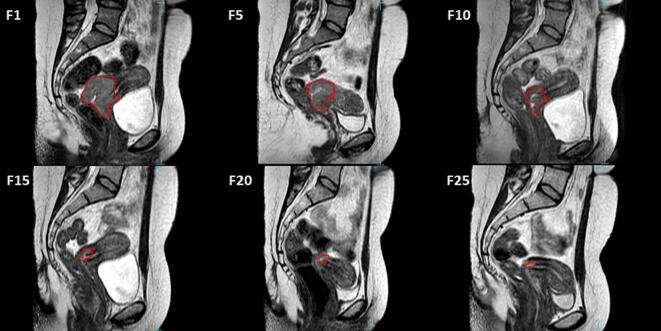
Example of tumor delineation for patient 10 over treatment for fractions (F) 1, 5, 10,15, 20, and 25.

Daily T2-weighted MRI scans were performed, using a 1.5 T MR-Linac system (Unity®, Elekta AB, Stockholm, Sweden), immediately before each treatment fraction. Gross tumor volumes (GTVs) were delineated retrospectively on these images (Fig. 1). The GTV was defined as hypersignal in the T2-weighted MRI and to ensure consistency and accuracy in tumor volume estimation, all delineations were reviewed and validated by two experts in the field of radiation oncology and cervical cancer.

### Tumor volume evolution analysis

Tumor volume evolution was analyzed throughout the course of radiotherapy. Thus, for each patient, tumor volumes were normalized relative to the initial volume at the first treatment fraction according to the following formula:

$$V_{\mathrm{norm},i}=\frac{V_{i}}{V_{1}}\times 100$$

Where *V*_i_ is the tumor volume at fraction i, and *V_1_* the initial volume.

Normalized tumor volumes were plotted for each patient throughout all the treatment fractions. A three-fraction moving average was applied to smooth any possible contouring inter-fractional variability. Treatment outcome was assessed at three clinically relevant timepoints. First, early response to EBRT was defined as achieving a ≥ 90% tumor volume reduction at fraction 25 (last treatment fraction). Second, tumor volume on pre-brachytherapy MRI was estimated using an ellipsoidal approximation based on the maximum tumor diameters measured in the axial, sagittal, and coronal planes on T2-weighted images, as part of routine clinical evaluation by experienced radiologists. Finally, the overall treatment response was evaluated on MRI acquired three months after brachytherapy, categorized as CR (complete response) or PR (partial response).

In addition, the time to 50% tumor volume reduction (T50) was calculated for each patient and defined as the number of fractions from treatment start to the first fraction at which the normalized tumor volume fell below 50% of the baseline value (V_norm, i_ ≤ 50%).

The tumor volume reduction rate (TVRR) was calculated at early treatment fractions to quantify the overall tumor evolution using the formula:

$${\mathrm{TVRR}}_{i}=\frac{V_{i}-V_{1}}{V_{1}}\times 100$$

To identify the earliest fraction providing robust predictive information, TVRR at multiple early treatment fractions (F5, F8, F10, F12, F15) was correlated with treatment response.

### Impact of treatment interruptions in tumor shrinkage

**Fig. 2:**
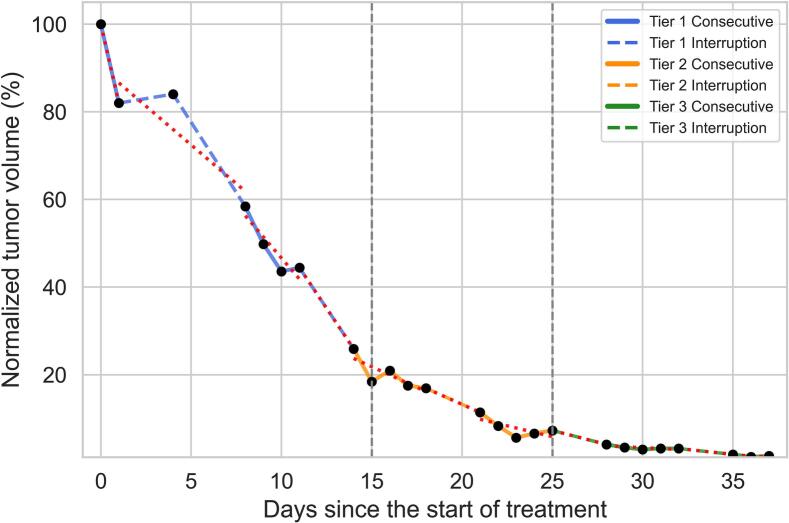
Example of treatment tiers and regression slopes calculation in Patient 10. Solid lines represent consecutive treatment intervals; dashed lines indicate interruptions (> 1 day). Regression lines fitted to each tier were used to calculate tumor volume reduction slopes.

To explore the effect of treatment interruptions (e.g. weekends or holidays) on tumor shrinkage dynamics and treatment response, treatment was divided into three phases: early (fractions 1–8), middle (fractions 9–17), and late (fractions 18–25). Inter-fraction intervals were classified as consecutive (1-day interval) or interrupted (>1-day interval, e.g. weekend/holidays). If an interval included fractions from two phases, it was assigned to the phase containing the median fraction number of that interval. An example on one patient is shown in Fig. 2.

### Statistical analysis

Descriptive statistics were used to summarize patient demographics, tumor characteristics, and treatment parameters. Tumor response dynamics were analyzed using two metrics: normalized tumor volume reduction rate (TVRR), and time to 50% tumor volume reduction (T50). Slopes of normalized tumor volume change (%/day) were estimated using linear regression for each inter-fraction interval and compared between consecutive and interrupted intervals within each treatment phase using the Wilcoxon rank-sum test.

Spearman correlation coefficients were used to assess the relationship between early TVRR, T50, and treatment response. A two-sided p-value < 0.05 was considered statistically significant. All analyses were performed using Python (version 3.11).

## Results

### Patients characteristics

From December 2021 to July 2025, 22 LACC patients underwent radiotherapy on the Unity MR-Linac. Sixteen consecutive patients that were meeting the inclusion criteria for this retrospective study and were included in the final analysis. Patients clinical and treatment characteristics are summarized in Table 1. A total of 400 MR images were analyzed.

**Table 1:** Patients’ and treatment characteristics.

Characteristics		Values
Age (years)		
	Median (range)	53 (26–94)
Histology, N (%)		
	Squamous cell carcinoma	14 (87.5)
	Adenocarcinoma	2 (12.5)
HPV status, N (%)		
	HPV+	14 (87.5)
	HPV-	2 (12.5)
FIGO stage, N (%)		
	IB2	3 (18.75)
	IIA2	1 (6.25)
	IIB	6 (37.5)
	IIIC1	6 (37.5)
Chemotherapy cycles, N (%)		
	None	2 (12.5)
	2	2 (12.5)
	4	1 (6.25)
	5	10 (62.5)
	6	1 (6.25)
Nodal involvement, N (%)		
	Negative	10 (62.5)
	Positive	6 (37.5)
Radiotherapy dose, N (%)		
	45 Gy in 25 fractions	10 (62.5)
	45/55 Gy in 25 fractions	6 (37.5)

The median age of the patients was 53 years (range 26–94 years). Most patients had squamous cell carcinomas (n = 14, 87.5%), while 2 patients (12.5%) had adenocarcinomas. Human papillomavirus (HPV) infection, assessed by P16/18 expression, was confirmed in 14 patients (87.5%) and absent in 2 patients (12.5%).

Patients were distributed across FIGO stages as follows: 10 (62.5%) were classified as stages IB–IIB (IB2 and IIA2), and 6 (37.5%) as stage IIIC1. Pelvic nodal involvement was identified in 6 patients (37.5%).

The median interval between the completion of MR-Linac-based EBRT and the first session of brachytherapy was 18.5 days (IQR, 12.5–20.5 days; range, 5–22 days).

### Tumor volume evolution

**Fig. 3:**
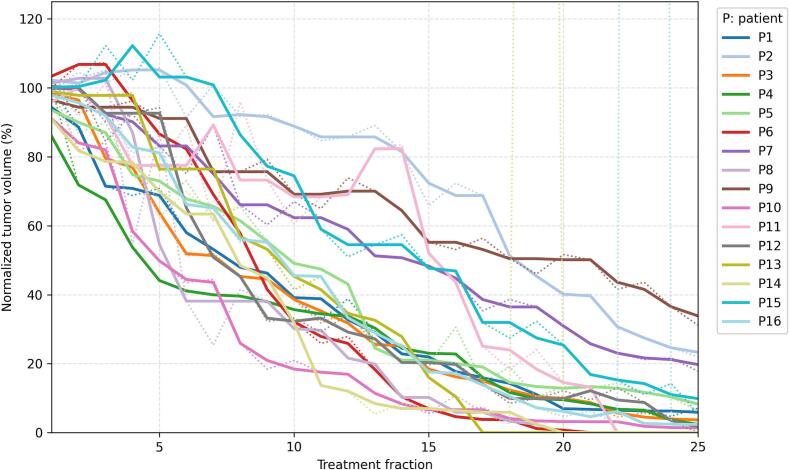
Per-fraction normalized tumor volume evolution throughout radiotherapy treatment. Dotted lines represent the raw normalized tumor volumes for each patient, while solid lines show the three-fraction moving average used to smooth fluctuations possibly caused by delineation inaccuracies.

The median tumor volume at baseline and at the end of EBRT was 26.2 cc (range 3.4–78.8 cc) and 0.7 cc (range 0 – 6.5 cc), respectively. Individual normalized tumor volume shrinkage trajectories are shown in Fig. 3.

**Fig. 4:**
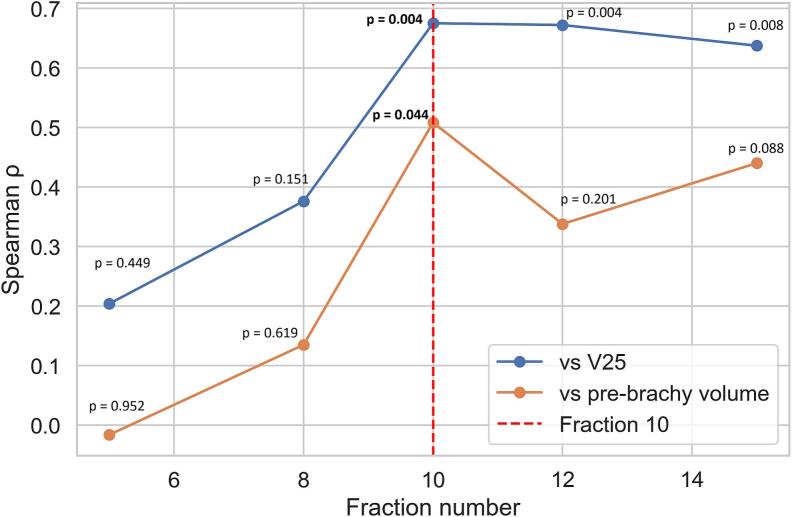
Spearman correlation coefficients (ρ) between early tumor volume reduction rates and tumor volume at fraction 25 (V25, blue) and pre-brachytherapy residual tumor volume (orange), evaluated at different treatment fractions. The vertical dashed line indicates fraction 10, corresponding to the time point with the strongest association with final EBRT response. (For interpretation of the references to colour in this figure legend, the reader is referred to the web version of this article.)

Spearman correlation coefficient between early TVRR (treatment fractions F5, F8, F10, F12, F15) and volume at fraction 25 increased progressively over the first 2 weeks, from ρ = 0.2 at fraction 5 (p = 0.45) to ρ = 0.38 at fraction 8 (p = 0.15), and peaked at fraction 10 (ρ = 0.67, p = 0.004) (Fig. 4). All patients with a tumor volume reduction greater than 50% by fraction 10 achieved a ≥ 90% reduction by the end of EBRT. A similar pattern was shown for the correlation with pre-brachytherapy response, with spearman correlation coefficients increasing from ρ = -0.02 (p = 0.95) at fraction 5 to ρ = 0.51 (p = 0.04) at fraction 10 (Fig. 4). No significant correlation could be observed with 3-month post EBRT response in this cohort.

T50 metric also differed significantly between response groups at the end of EBRT. Good responders reached a 50% reduction earlier (median 8 fractions) compared with poor responders (median 19 fractions; p = 0.017).

### Impact of treatment interruptions on tumor volume evolution

**Fig. 5:**
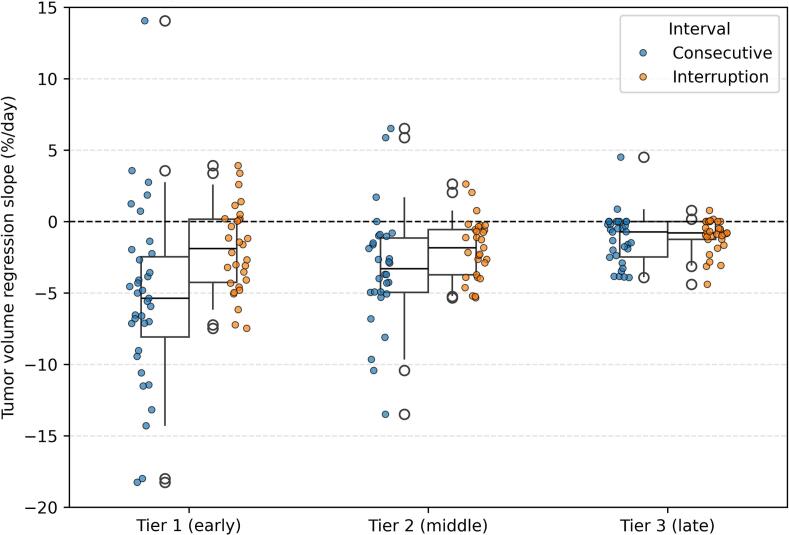
Comparison of tumor volume regression slopes (%/day) during consecutive versus interrupted treatment intervals across three treatment phases (tiers).

As shown in Fig. 5, tumor shrinkage rates were faster during consecutive treatment intervals compared to interrupted intervals, particularly in the early phase of the treatment course. In tier 1 (early phase), the median tumor volume regression slope was significantly steeper during consecutive intervals (−5.38% /day,) compared to interrupted intervals (−1.62% /day, p = 0.003, Wilcoxon rank-sum test). This difference became less present in the middle phase (tier 2: −3.58%/day vs −2.00%/day, p = 0.083) and was absent in the late phase (tier 3: −1.41%/day vs −0.88%/day, p = 0.271).

## Discussion

This study demonstrates that early tumor volume reduction, particularly by the 10th fraction of radiotherapy, is strongly associated with final EBRT tumor regression in patients with LACC. These findings are consistent with previous studies, which have suggested that early volumetric response during treatment is a promising predictive biomarker of outcomes in cervical cancer [14], [16], [17]. Consistent with our findings, Ohtaka et al. [16] reported in a cohort of 254 patients that a mid-EBRT tumor volume reduction ratio < 68.8% in tumors ≥ 34.1 cm^3^ was an independent predictor of poor outcome (HR for OS 3.10; 95% CI 1.57–6.15; p = 0.001). Similarly, Sun et al.[14] demonstrated that TVRR and mid-radiotherapy tumor volume are strong prognostic parameters for patients with locally advanced cervical cancer receiving concurrent chemoradiotherapy (CCRT).

Early volumetric response has also shown prognostic values in other tumor sites, although methodologies and imaging schedules vary across studies. In particular, Bernchou et al. [18] found results similar to ours in glioblastoma as they demonstrated that GTV shrinkage measured as early as fraction 10 strongly correlated with post-treatment outcomes defined as three weeks after the end of radiotherapy (R = 0.74, p < 0.001).

In rectal cancer, Fiorino et al. [19] and later Cusumano et al. [20] validated the Early Regression Index (ERITCP), showing strong predictive performance for pathological complete response and long-term clinical outcomes. These studies demonstrated that early regression captured at a mid-treatment time point carries biological and prognostic significance. They found that 25 Gy was the best biologically effective dose (BED) level to perform predictions (AUC = 0.93). However, ERITCP relies on two discrete MRI scans (pre-treatment and mid-treatment)**,** which limits the temporal resolution of the tumor response. Our study extends this concept by using daily MRI, enabling continuous assessment of tumor regression.

Recent cervical cancer studies have shown that low residual tumor volume on post-treatment MR imaging often corresponds to minimal or absent tumor cells on pathology, suggesting that volumetric response may reflect true histologic remission [21], [22].

However, to our knowledge, this is the first study to assess daily tumor volume evolution throughout the entire course of radiotherapy for cervical cancer patients. By leveraging novel MR-Linac data, we were able to perform a detailed and continuous assessment of tumor dynamics. This offers new opportunities for early prediction of response. The integration of volumetric response data into clinical decision-making opens the door for a new adaptive strategy. In settings where daily MR-guided radiotherapy is not available, an additional simulation CT scan around fraction 10 of the treatment could be considered in cervical cancer patients with large tumors undergoing EBRT to potentially adapt the treatment plan. However, this approach should be used with caution as tumor shrinkage may be associated with increased uterine mobility and reduced soft-tissue contrast on CT, which could limit accuracy of target delineation.

Brachytherapy remains the standard boost modality in locally advanced cervical cancer. However, it is an invasive procedure that can be heavy for the patients. Recent studies have demonstrated the feasibility and potential benefits of MR-guided SBRT boosts [23], [24], [25], providing high-precision dose delivery with good normal tissue sparing, especially when brachytherapy is not possible. Our findings support the potential for patient stratification based on early volumetric response. Patients exhibiting significant tumor regression by fraction 10 could be candidates for a stereotactic body radiotherapy (SBRT) boost delivered directly on the MR-Linac, especially when brachytherapy is not feasible. This could make the treatment easier on the patients, and improve their comfort. On the other side, patients with suboptimal early response may benefit from continuing with standard brachytherapy, which remain essential for achieving durable local control in radioresistant disease. However, this requires further clinical validation.

Thanks to the daily availability of MR images, we quantified the impact that treatment interruptions have on tumor shrinkage in the initial part of radiotherapy. In particular, we found the median tumor volume regression slope to be significantly steeper when fractions are delivered in consecutive days compared to interrupted intervals. These findings open the way for further studies to clarify the importance of treatment continuity in the early phase of treatment.

Emerging imaging biomarkers could further enhance early adaptive strategies. Recent data by Skipar et al. [26] suggest that combining hypoxia imaging at diagnosis with volume reduction measured at brachytherapy improves prognostic accuracy compared to either factor alone. Correlating hypoxia imaging of the tumor with early tumor response on day 10 could allow for an earlier and better personalized management of cervical cancers.

Our analysis had some limitations. Our sample size was limited. However, the limited number of patients is offset by the density of the daily longitudinal data. By using daily MR-Linac imaging, we achieved a temporal resolution that allowed us to identify significant regression trends. Minor inconsistencies occurred towards the end of treatment as the tumors shrank and became less visible on MRI. Despite this limitation, meaningful associations between tumor volume evolution and treatment response could still be identified. Additionally, tumor shrinkage is only one aspect of the biological response to radiation. Future studies incorporating multiparametric MRI and radiomic features could provide a more comprehensive understanding of tumor biology and help refine predictive models.

## Conclusions

MR-guided radiotherapy enabled a continuous, per-fraction assessment of tumor response in cervical cancer. Early tumor shrinkage, particularly by fraction 10, was strongly associated with final EBRT response, supporting its value as an early predictive marker. In addition, treatment interruptions were associated with significantly slower tumor regression during the initial phase of radiotherapy, highlighting the importance of treatment continuity early in the course.

These findings were made possible by the availability of daily high-quality MRI throughout treatment, allowing detailed characterization of individual tumor regression dynamics. Together, these results support the potential role of daily MR-guided volumetric monitoring to identify early responders and help personalized radiotherapy strategies. Validation in larger cohorts is warranted.

## Data sharing statement

The datasets generated and analyzed during the current study are available from the corresponding author on reasonable request, subject to institutional and patient confidentiality regulations.

## CRediT authorship contribution statement

**Perla Zidane:** Writing – original draft, Visualization, Methodology, Investigation, Formal analysis, Data curation. **Thomas Berger:** Writing – review & editing, Supervision, Methodology, Conceptualization. **Angela Boros:** Writing – review & editing, Resources, Conceptualization. **Ionela Caraivan:** Writing – review & editing, Resources. **Mazen Moussallem:** Writing – review & editing, Supervision, Methodology. **Corina Udrescu:** Writing – review & editing, Supervision, Methodology, Conceptualization. **Olivier Chapet:** Writing – review & editing, Supervision, Project administration, Methodology, Funding acquisition, Conceptualization.

## Funding

This work was conducted as part of a doctoral program funded by Elekta.

## Declaration of Competing Interest

The authors declare the following financial interests/personal relationships which may be considered as potential competing interests: Perla Zidane reports financial support was provided by Elekta. Thomas Berger reports financial support was provided by Elekta. Olivier Chapet reports financial support was provided by Elekta. Olivier Chapet reports a relationship with Elekta that includes: funding grants. If there are other authors, they declare that they have no known competing financial interests or personal relationships that could have appeared to influence the work reported in this paper.
